# Serum 14-3-3β protein: a new biomarker in asthmatic patients with acute exacerbation in an observational study

**DOI:** 10.1186/s13223-021-00608-4

**Published:** 2021-10-09

**Authors:** Decai Wang, Lizong Rao, Yalan Cui, Guoting Tang, Haiming Huang, Ting Yuan, Biwen Mo

**Affiliations:** 1 Department of Respiratory and Critical Care Medicine, Guangxi Zhuang Autonomous Region Education Department Key Laboratory of Respiratory Diseases, Affiliated Hospital of Guilin Medical University, Guilin, Guangxi China; 2 Department of Anatomy, Guilin Medical University, Guilin, Guangxi China

**Keywords:** Asthma, Biomarker, 14-3-3β

## Abstract

**Background:**

The determination of systemic inflammatory markers is one of the important directions to study the pathogenesis of asthma and improve the diagnosis of asthma. Current studies have found that the 14-3-3 protein family subtypes interact with target proteins to participate in the pathogenesis of a variety of immune inflammatory diseases. However, studies on serum tyrosine3-monooxygenase/tryptophan5-monooxygenase activation protein β (14-3-3β) in asthma are scarce. This study aimed to assess the clinical significance of 14-3-3β in asthmatic patients.

**Methods:**

We recruited 54 asthmatic patients with acute exacerbation and 50 asthmatic patients with chronic persistent. The normal control group included 54 healthy individuals. Clinical characteristics, clinical indicators [fractional expiratory nitric oxide (FeNO), eosinophil count, forced vital capacity (FVC), percent of predicted FVC (FVC% predicted), forced expiratory volume in one second (FEV1), percent of predicted FEV1 (FEV1% predicted), the ratio of forced expiratory volume in one second to forced vital capacity (FEV1/FVC) and serum 14-3-3β levels were measured to compare among each group. Spearman’s rank correlation coefficient was used to evaluate the correlation between 14-3-3β and clinical indicators. Finally, Receiver-operating characteristic (ROC) curves analysis was used to determine the sensitivity and specificity of 14-3-3β.

**Results:**

Our results showed that median (interquartile range) of serum 14-3-3β concentration (ng/mL) in acute exacerbation group of asthma (41.18 [33.06–51.76]) was much higher than that in normal control group (24.99 [17.43–29.91]; *P* < 0.001) and chronic persistent group of asthma (25.88 [21.03–34.55]; *P* < 0.001). Spearman’s correlation coefficient shows that the serum 14-3-3β level was positively correlated with FeNO (r = − 0.292, *P* = 0.032) and peripheral blood eosinophil count (r = 0.328, *P* = 0.016), and was negatively related to FEV1/FVC (r = − 0.293, *P* = 0.031) in the acute exacerbation group of asthma. At the same time, the serum 14-3-3β level was also negatively associated with FEV1 (r = − 0.297, *P* = 0.036) in the chronic persistent group of asthma. ROC curve analysis comparing acute exacerbation group of asthma with normal control group demonstrated a significant (*P* < 0.001) AUC of 0.90 (95% CI 0.85–0.96).

**Conclusion:**

The serum 14-3-3β protein may become a potential biomarker in asthmatic patients with acute exacerbation.

**Supplementary Information:**

The online version contains supplementary material available at 10.1186/s13223-021-00608-4.

## Background

Asthma is a common chronic airway disease leading to shortness of breath, chest tightness, and cough [1]. The best known endotype of asthma is type-2-high asthma, characterized by airway and blood eosinophilia, and the presence of biomarkers that depend on the type 2 cytokine IL-13, such as exhaled nitric oxide (FeNO) [1]. FeNO, an important biomarker of type 2 airway inflammation, is also associated with risk of adverse asthma outcomes [2, 3]. Eosinophils are pivotal cellular effectors in the Type 2 inflammatory pathway and are thought to play a major role in maintaining long-term inflammation in asthma [3]. One study suggest that high blood eosinophil count (> 400 cells/μ0) was potentially at greater risk of future exacerbations regardless of current Global Initiative for Asthma (GINA) control status and should be counselled and monitored accordingly [4]. Symptoms fluctuate over time and can worsen and lead to respiratory failure during periods of exacerbation, which are often precipitated by viral upper respiratory tract infections or commonly by exposure to aeroallergens or air pollution. It affects more than 300 million people globally, including 26 million in the United States [5]. Asthma causes substantial health and economic burdens, with more than 0.19 deaths per 100,000 people in the world each year [6]. Currently, diagnosis and treatment of asthma are often based on symptoms and lung function test results [7]. However, these may not be able to predict future exacerbation. In addition, many primary care facilities are not equipped with spirometry to perform the necessary tests. Hence, identifying new biomarkers that can enhance the detection rate in patients with asthma has been a hot research topic.

The 14-3-3 protein family is constituted by 28–33 kDa acidic proteins [8] found in all eukaryotes that play a role in the regulation of intracellular functions including protein synthesis, cellular metabolism, protein trafficking, signal transduction, and cytoskeletal transport [9–11]. In mammalian cells, 14-3-3 protein has seven isoforms (α/β, ε, η, γ, σ, θ/τ, and δ/ζ), with α and δ representing the phosphorylated versions of β and ζ, respectively [12, 13]. The 14-3-3 protein family is a class of proteins able to interact with a multitude of targets by establishing protein–protein interactions (PPIs) [10]. Prior research has demonstrated that the misregulation of 14-3-3 proteins contributes to important human diseases such as cancer, neurodegenerative disorders, and infection by Giardia intestinalis [14–17]. However, Current studies have found that the 14-3-3 protein family subtypes interact with target proteins to participate in the pathogenesis of a variety of autoimmune disease including rheumatoid arthritis, systemic sclerosis, or large vessel vasculitis [18–20]. Furthermore, Li et al. [21] found that upregulating 14-3-3σ can enhance IgE class switching and antibody secretion of B cells in asthma model of miR146a overexpressing mice, which increases in IgE are linked with worsened asthma severity. More importantly, one study reported that 14-3-3β mRNA is overexpressed in rat asthma models [22].

Based on these findings, we hypothesized that 14-3-3β protein is elevated in asthmatic patients, especially in acute exacerbation of asthma. To test our hypothesis, we conducted an observational study to measure 14-3-3β levels in asthmatic subgroup and normal control group. Furthermore, the relationship of serum 14-3-3β with clinical indicators [FeNO, eosinophil count, forced vital capacity (FVC), percent of predicted FVC (FVC% predicted), forced expiratory volume in one second (FEV1), percent of predicted FEV1 (FEV1% predicted), and the ratio of forced expiratory volume in one second to forced vital capacity (FEV1/FVC)] was also analysed. Finally, in order to evaluate the diagnostic performance of 14-3-3β protein in patients with asthma, we performed ROC curve analysis between asthmatic subgroup and normal control group.

## Materials and methods

### Patients with asthma and controls

The present study was an observational study that included 104 consecutive patients who fulfilled the diagnostic criteria for asthma according to the Global Initiative for Asthma (GINA) [23], as shown in Additional file 1: Fig. S1. All patients were recruited from the Department of Respiratory and Critical Care Medicine, Affiliated Hospital of Guilin Medical University from June 2019 to October 2020. Patients with asthma were further classified according to Guidelines for bronchial asthma prevent and management in China [24] into acute exacerbation group with newly diagnosed asthma who had not yet received any treatment, including prednisone or inhaled corticosteroids and long-acting beta2 agonist bronchodilator combinations (ICS-LABA) and chronic persistent group with established diagnosed asthma who were receiving treatment of ICS-LABA. The acute exacerbation group of asthma was defined as the sudden onset of symptoms (wheezing, shortness of breath, coughing, chest tightness, etc.), or the exacerbation of the original symptoms. The chronic persistent group of asthma was referred to weekly occurrence of symptoms at various frequencies and/or degrees (wheezing, shortness of breath, chest tightness, coughing, etc.) according to Guidelines for bronchial asthma prevent and management in China. In addition, we recruited 54 healthy volunteers with matching age, and sex serving as a normal control group, and these were people who had visited the hospital for a routine check-up and who had no underlying disease. Exclusion criteria included: (1) acute respiratory infections within the last 4 weeks, such as pneumonia, acute and chronic bronchitis, and tuberculosis; (2) combined with other respiratory diseases, such as chronic obstructive pulmonary disease, bronchiectasis, and lung cancer; (3) severe liver, renal, and/or cardiac insufficiency; (4) pregnant or lactating women; (5) severe mental disorders; (6) patients with other comorbidities that might impact the results of the current study.

### Determination of FeNO level

The FeNO detector, Sunvou-CA2122, (Share Ltd. Wuxi, China) was used to detect the FeNO. Before testing, all participants were informed the details of the procedure, and the detection of FeNO was strictly followed as per the manufacturer’s instructions. The results were expressed in terms of parts per billion ppb (1 ppb = 1 × 10^−9^ mol/L).

### Lung function test

Pulmonary function tests were performed using a System 7 device (Minato Medical Science Co., Osaka, Japan) according to standard of the American Thoracic Society (ATS)/European Respiratory Society (ERS) [25]. The gender, age, height, and weight of all participants were entered into the machine, and the expected value was automatically calculated. Body mass index (BMI) was calculated as weight (kg) divided by height (cm) in squared meters. FVC, FVC% predicted, FEV1, FEV1% predicted, and FEV1/FVC were included for our analysis.

### Blood sample collection and analysis

Fasting blood samples were drawn, centrifuged and serum was placed in plain polystyrene tubes on the same day. Serum samples were sent to the laboratory for storage at − 80 °C. Peripheral blood eosinophil counts were performed using Sysmex XN2800 (Sysmex Co., Kobe, Japan) automatic blood cell analyzer on each participant. Serum concentrations of 14-3-3β in patients with asthma and controls were measured by ELISA kits according to the manufacturer’s instructions (CUSABIO Life Science Ltd., Wuhan, China).

### Statistical analysis

Statistical analysis was performed using SPSS software (version 20.0), Quantitative variables were presented as mean [standard deviation (SD)], which was for normally distributed data or as the median and 25th–75th percentiles [interquartile range (IQR)], which was for non-normally distributed data. Categorical variables were presented as frequencies and percentages. Distribution normality was established by the Shapiro–Wilk normality test. Differences of sex between different groups were tested by chi-square test. To compare more than 2 groups, the one-way analysis of variance (ANOVA) for normally distributed data (such as age) and Kruskal–Wallis test followed by the Mann–Whitney U test (if a statistical difference was observed for non-normally distributed variables) were used to determine whether statistical significance existed across the groups. The relationship of 14-3-3β to clinical indicators was assessed using Spearman’s rank correlation coefficient. Receiver-operating characteristic (ROC) curves analysis was performed to evaluate the diagnostic utility of serum 14-3-3β. Significance was achieved when *P* < 0.05.

## Results

### Clinical characteristics and clinical indicators for all participants

This case control study included 104 asthmatic patients. 54 cases were in the acute exacerbation group, with an age range between 19 and 66 years, and a median of 45 years, including 23 (43%) females and 31 (57%) males. 50 cases were in the chronic persistent group, with an age range between 18 and 66 years, and a median of 45 years, including 33 (66%) females and 17 (34%) males. 54 cases were in normal control group aged between 18 and 64 years with a median of 37, which included 30 females (56%). Clinical characteristics (age, gender, and BMI) and clinical indicators (FeNO, eosinophil count, FVC, FVC% predicted, FEV1, FEV1% predicted, and FEV1/FVC) for all participants are provided in Table 1. There was no significant difference in gender and age between the each group (*P* > 0.05).

**Table 1:** Participant’s characteristics and the level of clinical indicators

Group, n	Normal control group (54)	Chronic Persistent group (50)	Acute Exacerbation group (54)
Sex (M/F), n (%)	24 (44)/30(56)	17 (34)/33 (66)	31 (57)/23(43)
Age [median (IQR), years]	37.50 (26.00–49.00)	45.50 (32.75–54.00)	45.50 (31.00–53.25)
BMI (mean ± SD, kg/m^2^)	22.54 ± 2.64	22.54 ± 2.99	23.30 ± 3.64
FeNO [median (IQR), ppb]	15.00 (12.25–20.75)	38.00 (23.50–60.00)*	67.00 (43.00–104.50*^#^
Peripheral eosinophil count [median (IQR), 4 × 109/L]	0.09 (0.06–0.17)	0.29 (0.16–0.47)*	0.45 (0.24–0.60)*^#^
Serum 14-3-3β [median (IQR), ng/mL]	24.99 (17.43–29.91)	25.88 (21.03–34.55)	41.18 (33.06–51.76*^#^
FVC (mean ± SD, L)	3.37 ± 0.77	2.92 ± 0.65*	3.20 ± 0.96
FVC% pred (mean ± SD, %)	92.60 ± 11.28	93.59 ± 13.80	86.64 ± 17.24*^#^
FEV1 (mean ± SD, L)	2.91 ± 0.69	2.16 ± 0.55*	2.23 ± 0.85*
FEV1% pred (mean ± SD, %)	94.04 ± 11.39	82.24 ± 17.55*	70.64 ± 19.70*^#^
FEV1/FVC (mean ± SD, %)	86.26 ± 5.36	74.10 ± 10.86*	69.16 ± 13.50*

Results are represented as n, mean with SD or median (IQR). Data were tested by χ^2^ testing, ANOVA, or Kruskal–Wallis test

*BMI* body mass index, *FeNO* fractional expiratory nitric oxide, *FVC* forced vital capacity, *FEV1* forced expiratory volume in one second, *FEV1/FVC* the ratio of forced expiratory volume in one second to forced vital capacity, *% pred* % predicted

*P < 0.05 versus normalcontrol group

^#^*P* < 0.05 versus chronic persistent group

### Serum 14-3-3β expression in asthmatic subgroup and control group

**Fig. 1:**
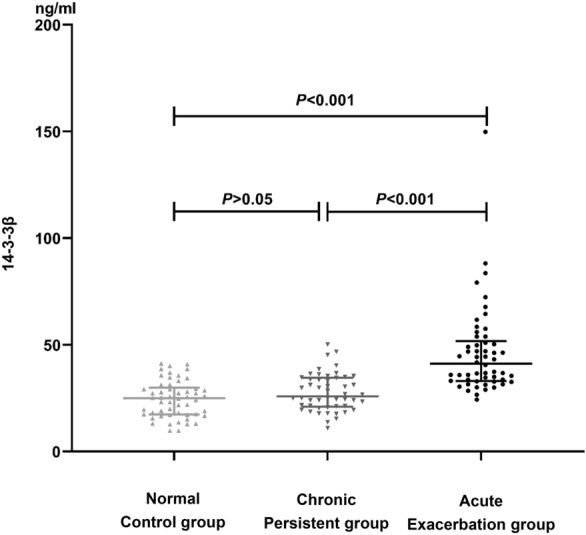
Comparison of serum 14-3-3β levels in normal control group and asthmatic subgroup

There was a statistically significant increase in the Median (IQR) 14-3-3β levels in the acute exacerbation group [41.18 ng/mL (33.06–51.76)] compared to the normal control group [24.99 ng/mL (17.42–29.91)], H = 7.46, *P* < 0.001, and the chronic persistent group [25.88 ng/mL (21.03–34.55)], H = 5.91, *P* < 0.001. However, there was no statistical heterogeneity in serum 14-3-3β level between the chronic persistent group and the normal control group (*P* > 0.05), as shown in Table 1 and Fig. 1.

### Correlation of serum 14-3-3β with clinical indicators in asthmatic subgroup

**Fig. 2:**
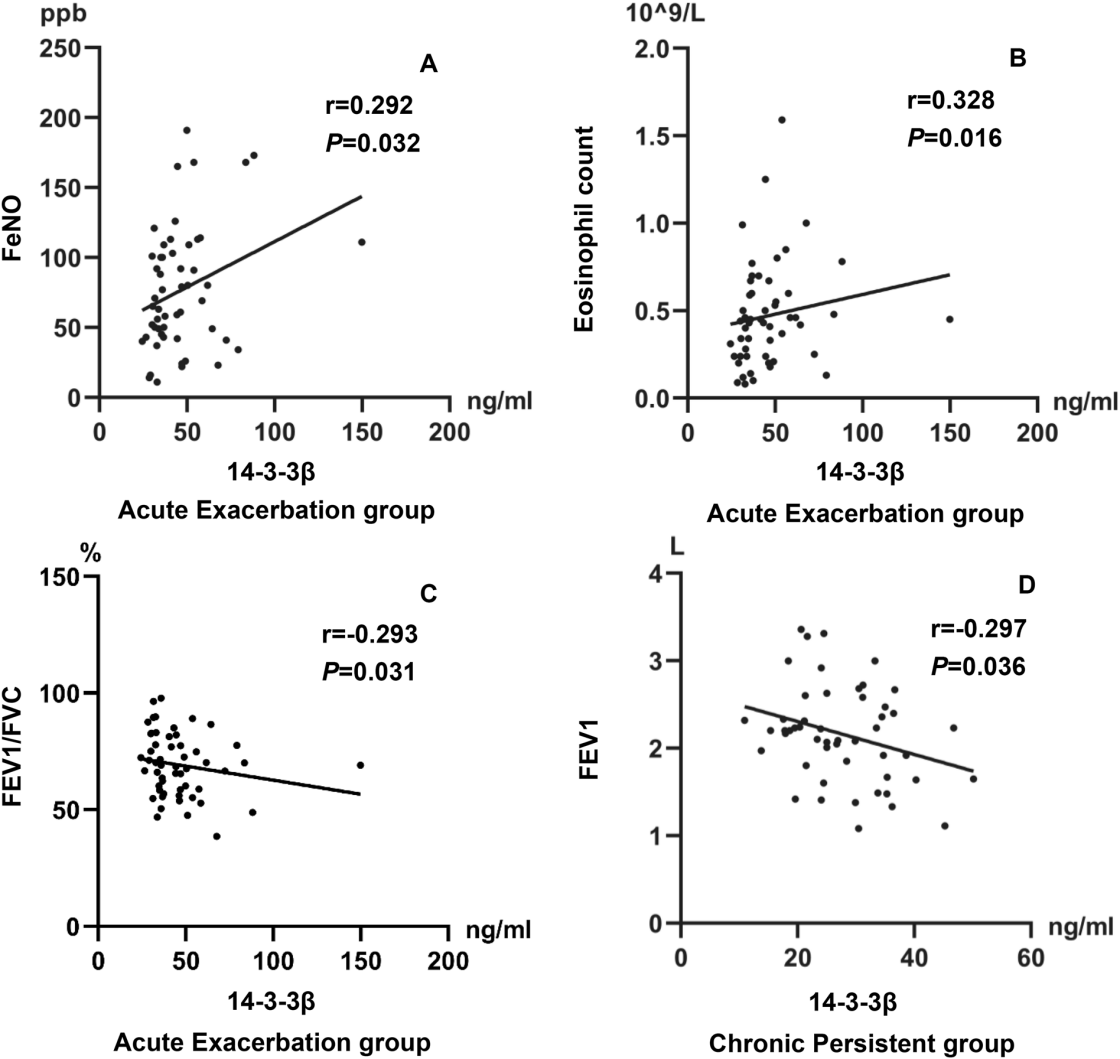
Correlation between serum 14-3-3β levels and clinical indicators in asthmatic subgroup. A-C. Correlation between 14-3-3β and FeNO; blood Eosinophil count; FEV1/FVC; FEV1/FVC% predicted in Acute Exacerbation group of asthma; D. Correlation between 14-3-3β and FEV1 in Chronic Persistent group of asthma

The correlation matrices presented in Table 2 illustrate the relationship between the levels of serum 14-3-3β and clinical indicators in the acute exacerbation group and the chronic persistent group, respectively. As expected, the levels of serum 14-3-3β was positively linked to FENO (r = 0.292, *P* = 0.032) and peripheral blood eosinophil count (r = 0.328, *P* = 0.016), and was negatively related to FEV1/FVC (r = − 0.293, *P* = 0.031) (r = − 0.299, *P* = 0.028) in the acute exacerbation group, as shown in Fig. 2A–C. Furthermore, the levels of serum 14-3-3β was also negatively associated with FEV1 (r = − 0.297, *P* = 0.036) in the chronic persistent group, as shown in Fig. 2D. However, correlation analyses of serum 14-3-3β concentration with FENO, peripheral blood eosinophil count, FVC, FVC% predicted and FEV1/FVC were not significant in the chronic persistent group. Furthermore, non-significant correlations were found among serum 14-3-3β concentration with FVC, FVC% predicted, FEV1 and FEV1% predicted in the acute exacerbation group.

**Table 2:** Spearman’s correlation coefficients between 14-3-3β and other clinical indices in asthmatic subgroup

	Acute Exacerbation group		Chronic Persistent group	
	Serum 14-3-3β (ng/mL)			
	*r*	*P*	*r*	*P*
FeNO (ppb)	**0.292**	**0.032**	− 0.192	0.190
Peripheral eosinophil count (4 × 109/L)	**0.328**	**0.016**	− 0.187	0.193
FVC (L)	0.072	0.605	− 0.166	0.251
FVC% pred (%)	0.039	0.780	0.156	0.280
FEV1 (L)	− 0.105	0.449	− **0.297**	**0.036**
FEV1% pred (%)	− 0.167	0.229	0.067	0.642
FEV1/FVC (L)	− **0.293**	− **0.031**	− 0.200	0.165

Bolding indicates statistical significance

*FeNO* fractional expiratory nitric oxide, *FVC* forced vital capacity, *FEV1* forced expiratory volume in 1 s, *FEV1/FVC* the ratio of forced expiratory volume in 1 s to forced vital capacity, *% pred* % predicted

### Receiver operating characteristic (ROC) curves of 14-3-3β in asthmatic subgroup

**Fig. 3:**
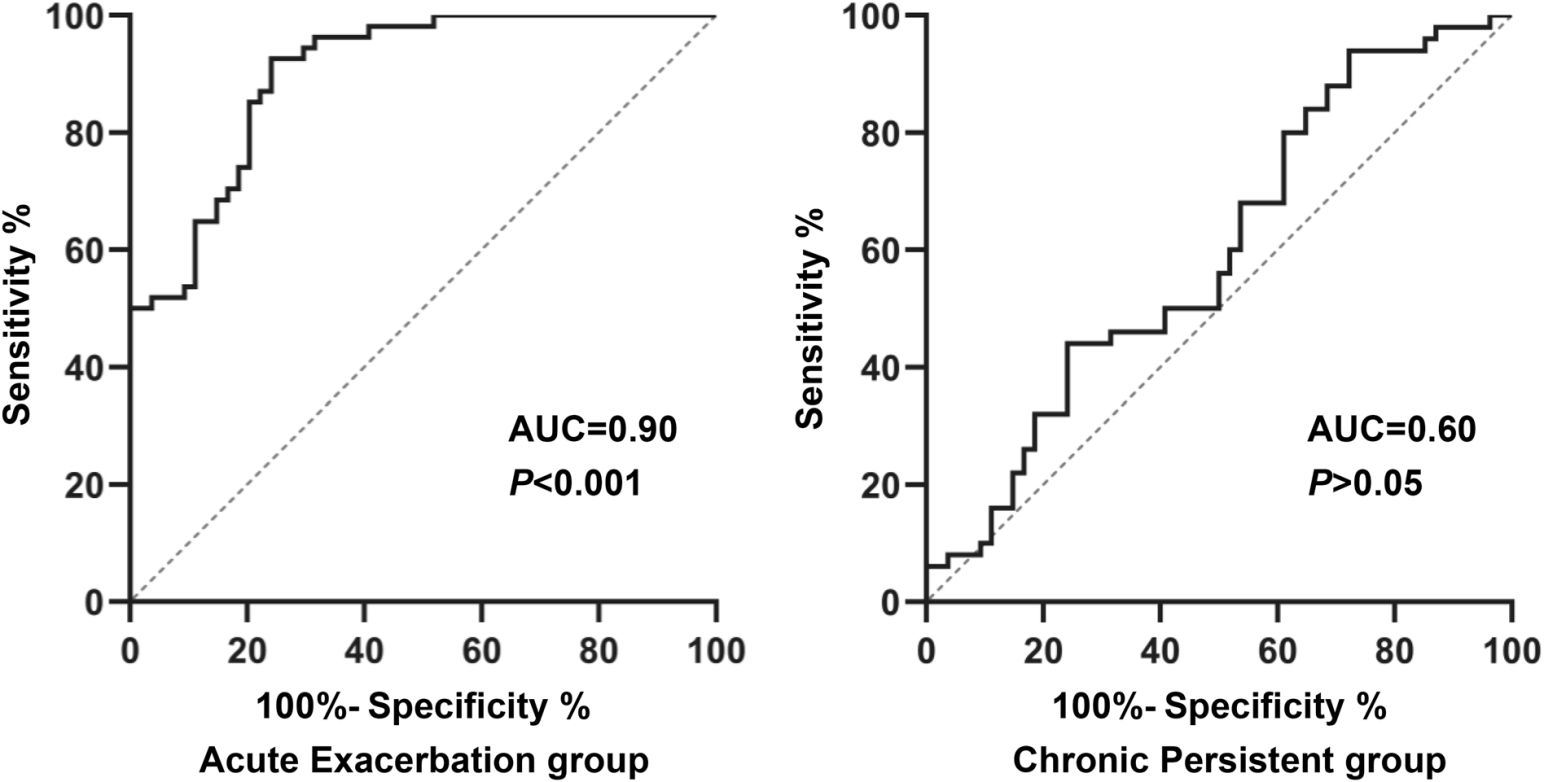
Receiver operating characteristic (ROC) curve for serum 14-3-3β levels comparing asthmatic subgroup with normal control group

ROC curve analysis comparing acute exacerbation group with normal control group demonstrated a significant (*P* < 0.001) AUC of 0.90 (95% CI 0.85–0.96), however, ROC curve analysis comparing chronic persistent group with normal control group demonstrated non-significant (*P* > 0.05) AUC of 0.60 (95% CI 0.49–0.70), as shown in Fig. 3 and Table 3. When the cut off value was 29.70 ng/mL, the ROC curve yielded a sensitivity of 92.6%, a specificity of 75.9%, a positive likelihood ratio (+LR) of 3.84, negative likelihood ratio (−LR) of 0.10 and a Youden index of 0.69 in acute exacerbation group.

**Table 3:** Diagnostic values of serum 14-3-3β in asthmatic subgroup comparing with normal control group

	Acute Exacerbation group	Chronic Persistent group
	Serum 14-3-3β (ng/mL)	
AUC (95% confidence interval)	0.90 (95% CI 0.85–0.96)	0.60 (95% CI 0.49–0.70)
*P*	***P* < 0.001**	*P* > 0.05
Cut-off value	29.70 ng/mL	29.70 ng/mL
Sensitivity (%)	92.6	44.0
Specificity (%)	75.9	75.9
+LR	3.84	1.83
−LR	0.10	0.74
Youden index	0.69	0.22

Bolding indicates statistical significance

AUC: area under the curve; +LR: positive likelihood ratio; −LR: negative likelihood ratio

## Discussion

Asthma is a serious global health problem affecting all age groups, with increasing prevalence in many developing countries, rising treatment costs, and a rising burden for patients and the community [5, 26, 27]. Currently, diagnosis and treatment of asthma are often based on symptoms and lung function test results. However, these may not be able to predict future exacerbation. In addition, many primary care facilities are not equipped with spirometry to perform the necessary tests. Therefore, the identifying new modalities for the laboratory diagnosis of acute asthma exacerbations is of great significance.

Some studies have found that 14-3-3 protein can regulate activation of T cell [28], generation of Th2 cytokine [19] and IgE class switching and antibody secretion of B cells [21] by interactions with target proteins. As far as we are aware, this is the first study to explore the features of changes in serum levels of 14-3-3β in patients with asthma. An increase in the serum concentration of 14-3-3β in the acute exacerbation group compared to the control group suggests that 14-3-3β may participate in the development of an acute exacerbation of asthma, and may therefore have some value in monitoring the state and prognosis of an acute exacerbation of asthma. However, there was no statistical heterogeneity in the serum level of 14-3-3β between the chronic persistent group and the normal control group, which may be explained by the chronic use of ICS-LABA in the chronic persistent group. In a study of the mouse model of eosinophilic meningitis, dexamethasone was demonstrated to decrease the expression of 14-3-3β protein in the CSF and in brain meninges [29].

FeNO is a biomarker of Th2 inflammation and is reported to be associated with clinical control of eosinophilic inflammation and asthma [3, 30]. In the present study there was a significant difference in FENO levels between asthmatic patients and control groups with higher values in the former, which was consistent with previous studies of Shrestha and colleagues [31]. Additionally, the levels of serum 14-3-3β was positively linked to FENO in acute exacerbation of asthma, indicating that 14-3-3β may be related to the airway inflammation of acute exacerbation of asthma. Eosinophils are pivotal cellular effectors in the Type 2 inflammatory pathway and are thought to play a major role in maintaining long-term inflammation in asthma [3, 32]. In this study, asthmatic patients showed significant differences in blood eosinophil counts compared to the control group, which was in agreement with evidence from recent cross-sectional studies [32, 33]. Also, the levels of serum 14-3-3β was positively correlated with peripheral blood eosinophil count in acute exacerbation of asthma. The levels of serum 14-3-3β was positively correlated with peripheral blood eosinophil count in patients experiencing an acute exacerbation of asthma, providing further evidence that 14-3-3β may be related to the airway inflammation during acute exacerbations of asthma. While, there is no correlation in serum 14-3-4β levels with FENO and peripheral blood eosinophil count in the chronic persistent group, which may be affected by ICS-LABA use. In this study, asthmatic patients showed significant differences in FEV1, FEV1% predicted and FEV1/FVC compared to the control group, which was in agreement with previous studies of Cowan et al. [34]. Also, the levels of serum 14-3-3β was negatively related to FEV1/FVC in the acute exacerbation group and to FEV1 in chronic persistent group. These findings demonstrate that 14-3-3β is associated with airflow obstruction and indicate that it may be an important risk factor for lung function decline in asthmatic patients.

ROC curve analysis comparing the acute exacerbation group with the control group demonstrated a significant difference in AUC, however, AUC was not significantly different in the chronic persistent group compared to the control group. When the cutoff value was 29.70 ng/mL, the ROC curve yielded a sensitivity of 92.6%, a specificity of 75.9%, a positive likelihood ratio (+LR) of 3.84, negative likelihood ratio (−LR) of 0.10 and a Youden index of 0.69 in acute exacerbation group of asthma, suggesting that 14-3-3β might be as a novel marker for the diagnosis in acute exacerbation of asthma.

Our study has several limitations. Firstly, the sample size was relatively small to perform the necessary multi-factor analysis. A completely different longitudinal study is required to determine whether 14-3-3β can be used as a novel marker for the diagnosis in acute exacerbation of asthma. Secondly, we are unable to frequently measure 14-3-3β levels during the study period, which inhibits our ability to determine the effects of clinical intervention on 14-3-3β expression changes. Finally, due to incomplete data for evaluating asthma control, we did not perform asthma control test (ACT) scores on all asthmatic patients. Therefore, further research is needed to clarify the practical application value of 14-3-3β as a new biomarker in a clinical setting.

## Conclusion

An increase in the level of serum 14-3-3β protein may be associated with airway inflammation and impaired bronchial patency in the acute exacerbation of asthma. The serum 14-3-3β protein can be used to distinguish between asthmatic patients with acute exacerbation and healthy individuals and may become a potential biomarker in asthmatic patients with acute exacerbation.

## Supplementary Information

**Additional file 1. **Flow diagram of inclusion with asthmatic patients

## Data Availability

All datasets used and/or analyzed during the current study are available from the corresponding author on reasonable request.
