# Supplementary figures and images for: Serum 14-3-3β protein: a new biomarker in asthmatic patients with acute exacerbation in an observational study
Source: Allergy Asthma Clin Immunol. 2021 Oct 9;17:104. doi: 10.1186/s13223-021-00608-4 (PMC8502409; doi:10.1186/s13223-021-00608-4)

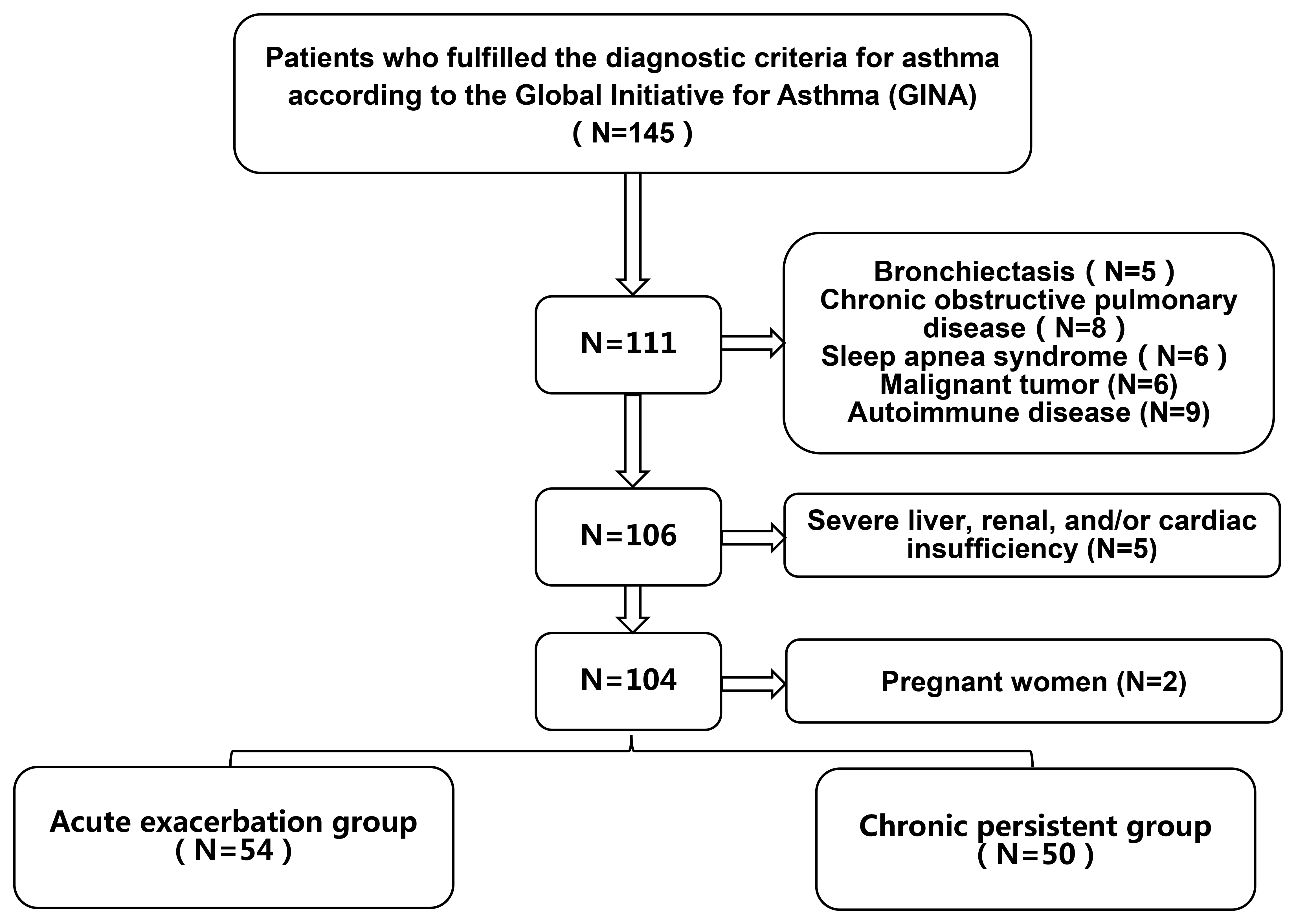

Supplement: Supplementary file 1 — Additional file 1. Flow diagram of inclusion with asthmatic patients [file 13223_2021_608_MOESM1_ESM.tif]
